# Smartphone-Linked and Electricity-Free Platforms for Rapid Colorimetric Molecular Detection of Poultry Respiratory Viruses at the Point of Need

**DOI:** 10.3390/bios15100638

**Published:** 2025-09-24

**Authors:** Mohamed El-Tholoth, Rabiha Seboussi, Mahmoud Hussein, Salameh Rahmdel, Alanoud Alalawi, Haim H. Bau

**Affiliations:** 1 Health Sciences Division, Al Ain Zakhir Campus, Higher Colleges of Technology, Abu Dhabi 17155, United Arab Emirates; h00348320@hct.ac.ae (S.R.); h00459153@hct.ac.ae (A.A.); 2 Department of Virology, Faculty of Veterinary Medicine, Mansoura University, Mansoura 35516, Egypt; 3 College of Veterinary Medicine, University of Al Dhaid, Sharjah, United Arab Emirates; rseboussi@uodh.ac.ae (R.S.); m.hussein@uodh.ac.ae (M.H.); 4 Department of Mechanical Engineering and Applied Mechanics, University of Pennsylvania, Philadelphia, PA 19104, USA; bau@seas.upenn.edu

**Keywords:** molecular detection, avian flu, H5N, ILTV, IBV, electricity-free, smartphone, colorimetric detection, LAMP, point-of-need

## Abstract

Efficient control measures for respiratory diseases in humans and farm animals require accurate, specific, and rapid diagnostics. Traditional PCR-based molecular diagnostics are restricted to centralized laboratories, which results in significant, potentially catastrophic delays in test results. A case in point is the recent avian flu outbreak, which has culled more than 280 million poultry birds worldwide (over 157 million in the USA alone) since 2022; has spread to other farm animals, such as cattle; has further heightened the risk of a human pandemic; and threatens food security. To enable molecular diagnosis of bird respiratory diseases at the point of need, we employ loop-mediated isothermal amplification (LAMP) in two platforms: (A) portable devices linked to a smartphone and (B) an inexpensive, disposable, electricity-free, instrument-free device with closed-tube, colorimetric detection that can be produced with minimal resources. Smartphone integration offers an unexplored opportunity for spatiotemporal disease mapping, equipping policymakers with critical data for outbreak control. Our assays demonstrated 100% sensitivity and specificity compared to the gold standard, lab-based, quantitative PCR (qPCR). We tested contrived samples of the avian flu H5N1 virus, laryngotracheitis virus (ILTV), and infectious bronchitis virus (IBV) spiked into clinical samples, achieving a detection sensitivity adequate for early infection diagnosis in under 45 min. The test is simple, requires minimal training, and can be performed without refrigeration, making it well-suited for resource-limited settings.

## 1. Introduction

Respiratory diseases spread rapidly among humans and animals, resulting in significant health and economic burdens. They are a leading cause of morbidity and mortality, responsible for approximately 4 million human deaths annually worldwide, with 80% occurring in developing countries [1,2]—nearly 14% among children [3,4]. This toll escalates during pandemics, such as COVID-19, which caused an estimated 14.9 million excess deaths globally between January 2020 and December 2021 [5].

The recent avian influenza (bird flu) has had significant global impacts, affecting the poultry industry, farm livestock, food security, and public health. In the United States alone, the 2022 avian flu outbreak led to the death or culling of more than 49 million birds, with estimated economic losses between $2.5 and $3 billion by the end of 2023 and a significant rise in egg prices for consumers [6,7]. The outbreak has also expanded beyond poultry, infecting farm mammals, such as dairy cows, and wild animals, such as big cats and domestic cats [8,9]. Additionally, several cases of human infection with avian influenza (H5N1) have been reported, primarily among dairy farm workers exposed to infected cattle [10], with the potential for mutations enhancing transmissibility remains a serious concern [11].

In addition to H5N1, other common pathogens, such as laryngotracheitis virus (ILTV) and infectious bronchitis virus (IBV), are highly contagious and cause respiratory diseases in chickens. Therefore, it is sensible to concurrently test multiple respiratory pathogens that produce similar clinical symptoms but require different control strategies.

Avian influenza virus (AIV) is a single-stranded, negative-sense RNA virus from the *Orthomyxoviridae* family. It infects farm animals, domestic animals, and occasionally humans. These zoonotic cases are linked to specific influenza subtypes, including H1N1, H2N2, H5N1, H7N7, and H7N9.

*Gallid alphaherpesvirus* 1 (GaHV-1), commonly known as infectious laryngotracheitis virus (ILTV), is a double-stranded DNA virus that causes infectious laryngotracheitis (ILT) disease. It is a member of the *Alphaherpesvirinae* subfamily of the *Herpesviridae* family. The infectious bronchitis virus (IBV) is a single-stranded RNA gamma-coronavirus that causes bronchitis infection [12,13,14,15,16,17,18,19].

In the absence of laboratory detection, clinical signs and post-mortem lesions assessment are used to make a tentative diagnosis of avian influenza virus (AIV), infectious laryngotracheitis (ILT), infectious bronchitis (IB), and other respiratory infections. Laboratory techniques include the hemagglutination inhibition (HI) test, enzyme-linked immunosorbent assay (ELISA), virus neutralization (VN) test, immunofluorescence techniques, quantitative real-time PCR (qPCR), and traditional PCR [15,17,19,20,21,22,23,24,25,26,27,28,29,30,31,32,33,34,35]. However, these diagnostic methods are time-consuming, require well-trained personnel, and rely on well-equipped laboratory facilities, which pose challenges for timely control measures. To address the need for rapid, specific, and sensitive diagnostics at the point of need, researchers, including our team, have developed loop-mediated isothermal amplification (LAMP) assays with sensitivity comparable to PCR for diagnosing respiratory viral infections in animals and humans [19].

Using a strand-displacing polymerase, the LAMP assay eliminates the need for high-temperature melting and thermal cycling required in PCR. Instead, LAMP operates at a constant temperature, typically 63–65 °C, simplifying equipment requirements [36,37,38,39,40,41,42]. Previously, we have shown that real-time fluorescence-based ILTV-LAMP and IBV-RT-LAMP assays detect and quantify pathogens’ genomes with sensitivity and specificity on par with qPCR [19,40,41].

Several commercially available incubators support LAMP-based diagnostics at the point of need, including the Genie II (Optigene, Horsham, UK), ESE-Quant TubeScanner (Qiagen, Lake Constance GmbH, Stockach, Germany), and EzDx WeD (Hangzhou, China, http://en.ezdxtech.com/, accessed on 3 June 2024). Recent research efforts have focused on developing field-portable diagnostic devices based on LAMP assays [43,44,45,46,47]. Isothermal amplification assays, such as LAMP, can be incubated using a simple temperature-controlled incubator, such as a water bath, or even without electricity. In electricity-free setups, heat can be generated via exothermic reactions involving magnesium alloys, calcium, or lithium with water or air. A phase-change material (PCM) buffers the temperature, maintaining a nearly constant, optimal incubation temperature for LAMP, regardless of ambient conditions. This strategy enables isothermal amplification to proceed without electricity, using minimal or no specialized equipment, and at low cost. The resulting systems are simple and robust and can be assembled virtually anywhere, independent of complex infrastructure or supply chains [48,49].

Smartphones are ubiquitous, making them suitable for point-of-need (PON) diagnostics with minimal or no extra cost. Smartphones can play a dual role in point-of-need devices. Smartphones can record test results with cameras, analyze test data, and transmit results to the cloud for real-time spatiotemporal surveillance [50].

**Figure 1:**
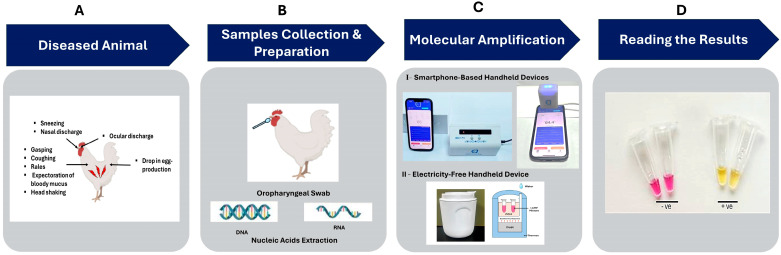
Workflow for molecular diagnosis of respiratory viruses. A symptomatic animal is identified (**A**); swabbed (**B**), and the sample is then lysed to release viral nucleic acids. These extracted nucleic acids are amplified using LAMP with the battery-powered smartphone-linked handheld devices ((**C**)—I) or electricity and instrument-free, chemically heated platform ((**C**)—II). The amplification process can be tracked with a colorimetric dye (**D**).

In this study, we use (A) a smartphone-linked, battery-powered handheld device and (B) an electricity-free platform for on-site colorimetric molecular detection of poultry respiratory viruses. Our systems rely on the loop-mediated isothermal amplification (LAMP) in a closed tube (Figure 1). One portable, smartphone-linked device accommodates two tubes, while the other eight tubes. The eight tubes can be used to test seven distinct samples for the same target and a control; analyze a single sample split across the tubes, each containing primers targeting a single target; to detect co-infections and positive and negative controls; or for a combination thereof. Our electricity-free platform processed four tubes concurrently. The number of test tubes can, however, be increased as needed.

## 2. Materials and Methods

### 2.1. Viruses

ILTV (Nobilis^®^ ILT, Intervet, Millsboro, DE, USA) and IBV (Vaxxon^®^ IB H120, IZO Vaxxinova, Brescia, Italy). The ILTV (strain PV/64) and IBV (strain Massachusetts H120) vaccines were generated through serial passages in embryonated chicken eggs (ECEs) and confirmed with qPCR as previously described [40,41]. The genome copy numbers were determined to be 2.5 × 10^4^ copies/μL for ILTV and 4 × 10^3^ copies/μL for IBV using a NanoDrop spectrophotometer (Thermo Scientific, Waltham, MA, USA) [29].

### 2.2. Synthetic cDNA Fragments of IV-Matrix Protein Gene

cDNA fragments based on a reference IV (H5N1) nucleotide sequence (Accession number: AM911067.1) (Supplementary Table S1) of the matrix protein gene were synthesized (GenScript, Piscataway, NJ, USA).

### 2.3. Spiked Clinical Samples

A total of 30 contrived samples (spiked oropharyngeal swabs) were used. The prepared samples included ten swabs containing ILTV, ten containing IBV, and ten swabs containing IV-cDNAs. Three negative samples were included. Oropharyngeal swabs were collected using sterile cotton swabs (Puritan™ 25-806 1WC FDNA, Guilford, ME, USA), each with an approximate absorption capacity of 100 μL. The swabs were placed into cryovials containing Dulbecco’s Phosphate-Buffered Saline (PBS) (Sigma-Aldrich, Steinheim, Germany).

### 2.4. Nucleic Acids Extraction

Nucleic acids were extracted from ILT and IB viruses using the AM1836 5X MagMax 96 Viral Extraction Kit (Life Technologies Corp., Carlsbad, CA, USA), following the manufacturer’s guidelines.

### 2.5. LAMP Primers

The primers were previously designed using PrimerExplorer V5 software (Eiken Chemical Co., Ltd., Tokyo, Japan) to target specific fragments of the polymerase protein-coding gene of ILTV (277 bp) and the nucleocapsid protein-coding gene of IBV (233 bp) [40,41]. Genomic sequences of various IV serotypes from GenBank were aligned and analyzed to identify conserved regions. A 236-nt sequence within the AIV matrix protein gene was selected as the template due to its high similarity across the analyzed serotypes. LAMP primers were designed accordingly (Supplementary Table S2). These primers were designed to avoid primer-dimer forming inter-complementary sequences and to avoid cross-reactivity with other chicken respiratory viruses, including Marek’s Disease virus (MDV), Newcastle disease virus (NDV), ILTV, and IBV based on BLAST analysis (NCBI database, version 2.14.1, http://www.ncbi.nlm.nih.gov, accessed on 20 December 2024). The LAMP primers were synthesized by IDT (Coralville, IA, USA) for ILTV and IBV and by GenScript (Piscataway, NJ, USA) for AIV, provided in lyophilized form, and reconstituted in nuclease-free water to a final concentration of 100 μM.

### 2.6. Smartphone Handheld Devices

We used two battery-powered EzDx WeD systems (Hangzhou, China, http://en.ezdxtech.com/, accessed on 3 June 2024) (Figure 1). The first device (EzDx WeD-1 Pro), measuring 139 mm × 58 mm × 75 mm and weighing 315 g, concurrently processes up to 8 reaction tubes. The second, more compact device (EzDx WeD-mini) accommodates two reaction tubes, measures 46 mm × 32 mm × 56 mm, and weighs 61 g. Both devices feature magnetic caps that secure the lid to the main body, applying pressure to the reaction tubes to enhance contact with the heating block and reduce thermal resistance.

The EzDx WeD devices support real-time fluorescence detection of the reactions’ amplicons and allow colorimetric detection. Through Bluetooth connectivity, both devices are controlled via smartphone applications (EzDx WeD-mini, Version 1.0.5, and EzDx WeD-1 Pro, Version 1.0.5; Hangzhou EzDx Technology Co., Ltd., Hangzhou, China, 2023), which feature an intuitive user interface that allows users to adjust temperature settings [51,52].

### 2.7. Electricity-Free Device

**Figure 2:**
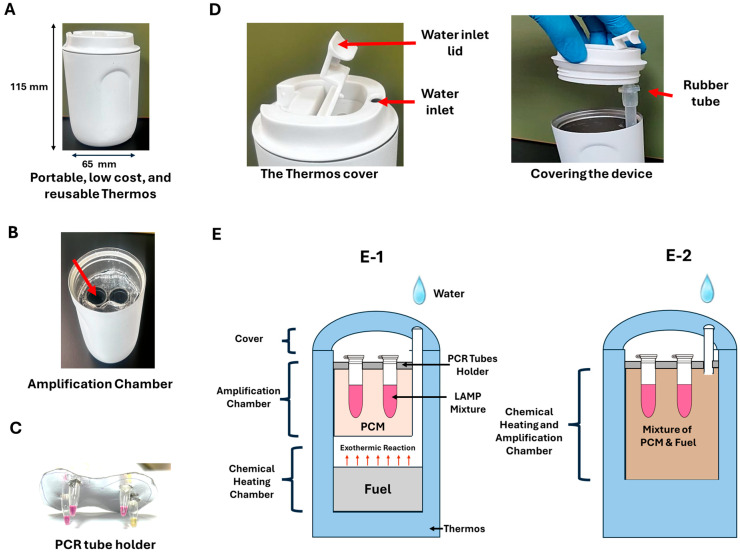
Our 115 × 65 mm electricity-free LAMP incubator (**A**) and its key parts (**B**–**E**). (**B**) The incubation chamber was filled with a phase-change material with a phase transformation temperature slightly above the LAMP incubation temperature. (**C**) The amplification chamber’s lid includes four bores to accommodate four test tubes (more are possible). (**D**) The Thermos lid has a water inlet. (**E**) A schematic diagram of the internal structure of our portable electricity-free device with the reaction tubes enveloped with PCM only (**E-1**) and a mixture of fuel and PCM for faster temperature ramp-up (**E-2**).

Our self-heated, electricity-free device is housed in a Thermos^®^ (Zhejiang Daian Commodity Co., Ltd., Jinhua, China) to provide thermal insulation (Figure 2). In our first embodiment, the bottom section of the Thermos contained an exothermic reaction chamber, like in [53]. The middle section held a phase change material (PCM) capped with an aluminum test tube holder measuring 50 mm in length and 25 mm in width. This holder contains pre-drilled four holes designed to securely accommodate the reaction tubes. As previously described, PCM and fuel are mixed in the second embodiment [49]. This latter configuration allows for faster temperature increases but does not permit PCM reuse. In contrast, the first embodiment enables PCM reuse. Below, we begin by describing the first embodiment.

A pack of Mg−Fe alloy (Innotech Products Ltd., Cincinnati, OH, USA) is placed at the bottom of the Thermos^®^ beneath the phase change material (PCM-A-64, Guangzhou Zhongjia New Material Technology Co., Ltd., Guangzhou, China). An exothermic reaction is triggered when water is added to the magnesium alloy, releasing heat [48].

$$\mathrm{Mg}(\mathrm{Fe})(s)+2\;H_{2}O\;(\ell )\to \mathrm{Mg}(\mathrm{OH}{)}_{2}\;(s)+H_{2}\;(g)\;\Delta H≅-14600\;\frac{Joules}{g-Mg}$$

In the above, (*s*), (*ℓ*), and (g) denote, respectively, solid, liquid, and gas. ΔH is the heat released by the reaction. The reaction tubes containing the LAMP mixtures are embedded entirely within the PCM, ensuring efficient heat transfer between the PCM and the reaction tubes.

The cover of the Thermos^®^ includes a water inlet opening with a cover lid that remains open after water is added, allowing the released hydrogen gas to escape. The water inlet is connected to a rubber tube that directs water into the exothermic reaction chamber.

When water containing 1% NaCl is added to the cup, it flows through the flexible tube and reacts with the Mg(Fe) alloy. This reaction increases the system’s temperature until it reaches the phase change temperature of the PCM. Beyond this temperature, any additional energy produced by the exothermic reaction is absorbed as latent heat, transforming the PCM from a solid to a liquid rather than increasing the system’s temperature. As a result, the system maintains the desired incubation temperature in a wide range of ambient temperatures. Once the exothermic chemical reaction is complete, the PCM transitions back from a liquid to a solid, releasing heat to prolong the time interval available for incubation. This process ensures our system maintains a nearly constant temperature for time intervals longer than needed for the LAMP amplification process without reliance on electricity and control circuits.

To reduce the temperature ramp-up time, we experimented with a second embodiment, wherein we prepared a homogeneous mixture of Mg/Fe powder (3 g) and PCM material (10 g). These components were placed in a 50 mL tube and thoroughly mixed for a few minutes using a vortex mixer (Cole-Palmer^®^, Quebec, QC, Canada), forming EPCM (Energetic Phase-Change Material) [49]. The mixture was then spread on Kimwipe paper and rolled, cigarette-like fashion, to create a “heating canister,” which was subsequently placed in the thermos (Figure 2(E-2)) (Supplemental Figure S1). The Kimwipe paper aids water transport through capillary action, counteracting the PCM’s hydrophobicity, which would otherwise hinder water infiltration and result in unreacted fuel pockets [54].

A K Type OMEGA Thermocouple (OMEGA Engineering^®^, Norwalk, CT, USA) was placed in one of the test tubes containing 25 μL of the LAMP mixture to evaluate the thermal performance of our electricity-free incubators. The tube’s temperature was monitored in real-time during experiments conducted at room temperature (22 °C), simulating the room temperature.

### 2.8. Fluorometric LAMP Assay

To verify our LAMP primers, templates of the ILTV polymerase gene, the IV matrix protein gene, and the IBV nucleocapsid gene were amplified, each with 2.5 μL of six specific LAMP primers (Supplement Table S2), 1.25 μL of 1× EvaGreen^®^ dye (Biotium, Inc., Fremont, CA, USA), 1.5 μL of extracted viral nucleic acid template, 12.5 μL of Isothermal MasterMix (ISO-001, OptiGene, Horsham, UK), 0.5 μL of AMV reverse transcriptase (200 U/μL) (Promega, Madison, WI, USA) only in the case of RNA targets, and nuclease-free water (Invitrogen, Carlsbad, CA, USA) to a total volume of 25 μL reaction mix.

The benchtop 7500-Fast Real-Time PCR thermal cycler (Applied Biosystems, Foster City, CA, USA), operating at a fixed temperature, was used to incubate the LAMP reaction and monitor intercalating dye fluorescence emission intensity during incubation at 65 °C. Each run contained non-template controls to confirm the absence of contamination and spurious amplification. The ILTV (for IV & IBV-RT-LAMP), IBV (for IV & ILTV-LAMP), MDV, and NDV (Intervet, Boxmeer, The Netherlands) vaccines were used to confirm specificity. All tests were performed in triplicate to assess reproducibility.

### 2.9. Colorimetric LAMP Assay

The LAMP reaction mix comprised WarmStart^®^ Colorimetric LAMP 2X Master Mix (DNA & RNA) (New England Biolabs Ltd., Ipswich, MA, USA). The 25 μL reaction mixture included LAMP primers, 12.5 μL of 2× Reaction Master Mix, one μL of viral nucleic acid template, and nuclease-free water to bring the total volume to 25 μL. Reactions were carried out at 65 °C for up to 40 min using EzDx WeD systems and electricity-free incubators. Polymerase byproducts included protons, lowering the reaction mix’s pH and causing a visible color shift in the phenol red pH indicator, included in the reaction mix, from pink to yellow, easily detectable by the naked eye (Figure 1D). Each run included template-free controls to ensure the absence of contamination and spurious amplicons. The assay’s specificity was confirmed as described earlier. All tests were performed in triplicate to assess reproducibility.

### 2.10. Analytical Sensitivity

We used ILTV, IV, and IBV-negative oropharyngeal swabs to perform 10-fold serial dilutions of ILTV DNA, IV-cDNA, and IBV RNAs. The fewest detectable genomic copies (analytical sensitivity) of ILTV, IV, and IBV were determined. Furthermore, we repeated the experiments eight times using 50, 100, 200, and 250 genome copies of ILTV; 50, 150, 250, 350, and 400 genome copies of IBV; and 5, 10, 25, and 50 genome copies of IV to identify the lowest detectable target quantity with 95% confidence. Probit analysis was performed using R software (version 3.6.3, R Development Core Team, https://www.r-project.org/).

### 2.11. Detection of Nucleic Acids from Spiked Clinical Samples

A total of 30 contrived samples were tested: 10 swabs containing ILTV, 10 containing IBV, and 10 containing IV-IV-cDNAs copies. All samples were analyzed using LAMP assays with smartphone-based devices and electricity-free devices. Concurrently, the same samples were tested with qPCR, using the LAMP F3 and B3 primers as PCR forward and reverse primers [35,40,41]. Negative controls were included in all our tests.

To evaluate the feasibility of detecting respiratory infections in chickens using a simple and cost-effective nucleic acid extraction approach, we tested six raw clinical chicken samples, three positives for ILTV and three positives for IBV after incubation at 95 °C for 5 min prior to adding each sample to the reaction mix.

## 3. Results

### 3.1. Benchtop LAMP Assays

To test the specificity of our LAMP primers, we incubated ILTV DNA (2.5 × 10^4^ gDNA copies/μL), IBV RNA (4 × 10^3^ gRNA copies/μL), IV cDNA (5 × 10^3^ cDNA copies/μL), NDV (10^2^ EID_50_/μL), and non-template control (NTC) with each of the ILTV, IV, and IBV primer sets separately. None of the off-target templates amplified within the incubation time of 45 min. ILTV-DNA with ILTV LAMP primers (threshold time 9 min), IBV RNA with IBV LAMP primers (threshold time 13 min), and IV-cDNA with IV LAMP primers (threshold time 8 min) produced distinct amplification curves. IBV RNA with IV LAMP primers, ILTV DNA with IBV LAMP primers, and IV-cDNA with IBV LAMP primers did not amplify (no false positives). Melting curves of each positive LAMP assay product exhibited a distinct peak, confirming the absence of non-specific amplificons and primer-dimer formation.

### 3.2. Smartphone-Linked, Handheld Devices

**Figure 3:**
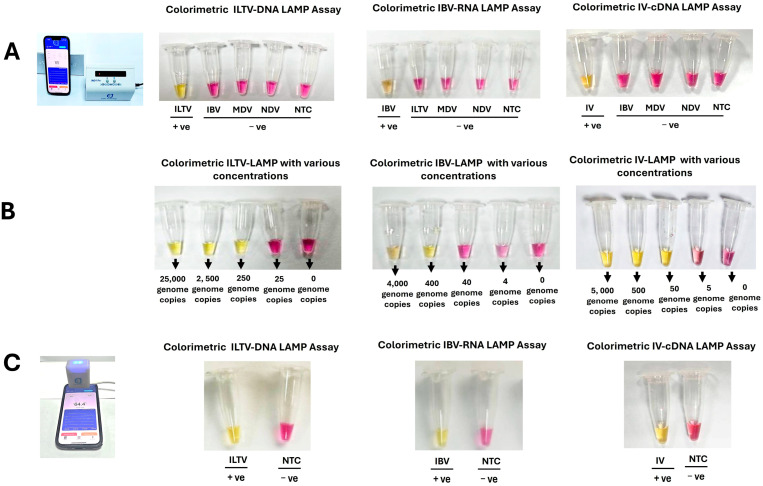
(**A**) Specificity. End-point colorimetric detection of ILTV, IBV, and IV with EzDx-Wed-1 Pro device (8 reaction tubes). Each sample comprised 2.5 × 10^4^ ILTV-gDNA, 4 × 10^3^ gIBV RNA, 5 × 10^3^, IV cDNA, 5 × 10^3^ MDV-gDNA, and 10^2^ EID_50_ NDV per reaction volume. Only reactions with target-matched primers exhibited color changes, indicating true positives. All off-target samples presented negative results within 30 min of incubation. (**B**) Sensitivity: The colorimetric assay successfully detected as few as 250 ILTV, 400 IBV, and 50 IV copies per reaction volume. (**C**) End-point colorimetric detection of ILTV, IBV, and IV with EzDx WeD-Mini device (2 reaction tubes).

We used the portable, small-footprint EzDx WeD-1 Pro (8 reaction tubes) and EzDx WeD-Mini (2 reaction tubes) devices to incubate our LAMP assays and visually inspect the solution color at the end of incubation (Figure 3). Each test included a non-template control to monitor false positives.

The colorimetric (pH) detection monitors proton production during polymerase amplification. When the target is absent (negative test)—the reaction mix remains alkaline and pink. During amplification, the reaction mix’s pH shifts to acidic, and the pH indicator changes color to yellow. Using this colorimetric detection, we observed 100% specificity (no false positives) within the 30 min incubation.

Our LAMP assays with the handheld EzDx devices yielded positive amplification with 250 copies of ILTV-DNA, 400 copies of IBV-RNA, and 50 copies of IV-cDNA. However, no color change from pink to yellow was observed at 25 DNA copies for ILTV, 40 RNA copies for IBV, and 5 cDNA copies for IV (Figure 3B). Probit analysis of our data (Supplementary Table S3) indicated that the analytical sensitivity of the LAMP assays, with 95% confidence, was estimated at 166 copies of ILTV -DNA, 265 copies of IBV-RNA, and 38 copies of IV. The sensitivity was on par with the handheld EzDx devices and the benchtop thermal cycler.

### 3.3. Electricity-Free Device

The EzDx Pro and EzDx Mini devices range in price from a few hundred to over a thousand U.S. dollars. To make our on-site respiratory disease tests affordable, we evaluated electricity-free, self-regulating, chemically heated incubators. Despite significant fluctuations in ambient temperature, the phase change material (PCM) maintained a stable temperature suitable for LAMP incubation [49]. All experiments were performed in triplicate and showed high reproducibility. At an ambient temperature of 22 °C, test tubes remained between 59 °C and 61 °C for approximately one hour, longer than required for LAMP amplification.

In our first embodiment of the chemical heater, the exothermic reaction chamber was physically separate from the PCM, resulting in a relatively long ramp-up time (~15 min). In our second design, we combined the reactants with the PCM to create a composite material, dubbed energetic phase change material (EPCM). This integration reduced the ramp-up time to under 5 min (Supplementary Figure S2). Both designs yielded comparable amplification results. In the EPCM format, however, the PCM is not reusable (without treatment) and must be discarded after use. Both the fuel (magnesium alloy) and PCM are inexpensive, costing only a few cents, and environmentally friendly. The overall cost can be further reduced by replacing the thermos container with a Styrofoam cup.

**Figure 4:**
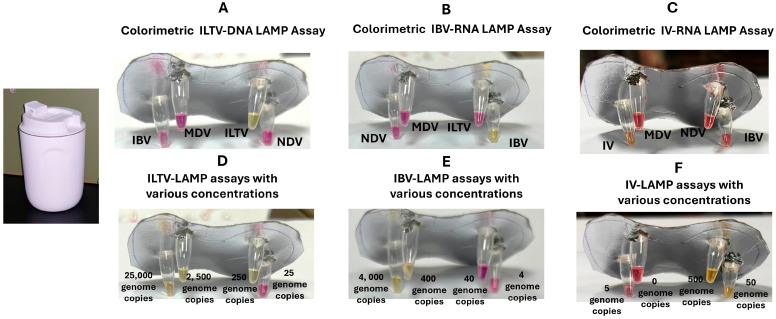
Our electricity and instrument-free devices exhibited no cross-reactivity (false positives) when detecting ILTV (**A**), IBV (**B**), and IV (**C**) calorimetrically. Colorimetric detections of LAMP ILTV (**D**), IBV (**E**), and IV (**F**) products as functions of template concentrations.

We used our electricity-free devices for colorimetric ILTV, IBV, and IV detection to demonstrate the feasibility of instrument-free molecular detection of respiratory pathogens at the point of need. In the presence of the target nucleic acids ILTV-DNA, IV-cDNA, and IBV-RNA at the indicated concentrations, the corresponding reaction mixtures changed color from pink to yellow (Figure 4). Our essays showed no cross-reactivity with any of the respiratory pathogens tested.

The colorimetric LAMP assays, when incubated using our electricity- and instrument-free devices, produced positive results with 250 copies of ILTV-DNA, 400 copies of IBV-RNA, and 50 copies of IV-cDNA (Figure 4D–F), comparable to the outcomes obtained with the EzDx devices. Probit analysis of the data (Supplementary Table S3) estimated the limit of detection (LOD) with 95% confidence to be 212 copies for ILTV-DNA, 268 copies for IBV-RNA, and 52 copies for IV.

### 3.4. Clinical Performance of ILTV, IBV, and IV LAMP Assays

We tested 33 contrived samples (oropharyngeal swabs spiked with the same virus concentrations, which gave positive results when diluted in nuclease-free water, as previously mentioned). Among these, 10 contained ILTV gDNA, 10 had IBV gRNA, 10 included IV-cDNA, and three served as pathogen-free negative controls (NC). All samples were analyzed using LAMP assays incubated with smartphone-linked EzDx devices and our electricity and instrument-free platform. Our LAMP test results were compared with the gold standard qPCR data (Supplementary Table S4).

**Figure 5:**
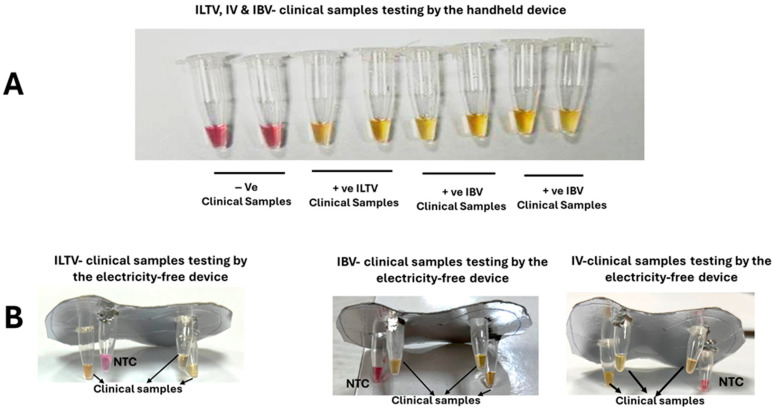
Colorimetric LAMP tests with EzDx Pro (**A**) and our electricity-free device (**B**) of oropharyngeal swabs spiked with ILTV, IV, and IBV targets.

All samples that tested positive for ILTV (N = 10), IBV (N = 10), and IV (N = 10) by q(RT)PCR also tested positive with the EzDx devices and our homemade electricity-free platforms, and all negative controls presented negative test results (Figure 5).

Lastly, we tested oropharyngeal swabs spiked with ILT and IBV whole viruses. The samples were pre-heated to 95 °C for 5 min prior to their introduction into the LAMP reaction mix. This approach yielded positive results within 45 min. To verify the specificity of their amplification, the samples were tested again using the fluorometric LAMP assay as described above. A single peak was generated from melting curve analysis of the positive LAMP products, confirming the absence of non-specific amplification.

## 4. Discussion and Conclusions

Poultry and eggs are vital components of diets in both developing and developed countries, offering high-quality protein, essential vitamins, and minerals at relatively low cost [55]. Respiratory diseases in poultry are highly contagious and spread rapidly among birds and other animals. Recent outbreaks of avian influenza (H5N1 and other strains) have highlighted the severe global consequences of such diseases, affecting not only the poultry industry but also food security, public health, and national economies [19].

In developed countries, animal diseases are diagnosed by collecting samples from suspected diseased animals and shipping them to centralized, well-equipped laboratories. This process typically has a turnaround time of a few days from sample collection to test results, which can delay the implementation of control measures. The situation is even more challenging in developing countries, where resource constraints often make molecular diagnostics unavailable, further hindering timely disease identification and control. Both developed and developing countries would benefit from inexpensive, on-site molecular diagnostics that can be performed by minimally trained personnel, delivering test results within an hour of sample collection and enabling real-time control measures.

To address these needs, we examined the feasibility of performing molecular testing at the point of need with small, inexpensive, smartphone-linked portable devices and with homemade, electricity-free, disposable incubators. We selected Loop-Mediated Isothermal Amplification (LAMP) with colorimetric amplicon detection. LAMP does not require thermal cycling, simplifies incubators, reduces power consumption, and enables electricity/instrument-free operation. These devices generally range in price from a few hundred to a thousand US dollars [51]. Furthermore, LAMP is highly prolific, producing more amplicons than PCR, which offers many, including colorimetric, methods to detect polymerase amplicons and byproducts [19]. We used LAMP primers developed by our group for IBV [40], ILV [41], and IV. Despite being inexpensive, portable, and compact, the devices deliver performance comparable to handheld smartphone-based devices. Our point-of-need assays performed as well as the far more expensive and complicated benchtop equipment, providing equivalent sensitivity and selectivity.

The EzDx devices are linked to the ubiquitous smartphone that controls device operation and reports test results. The smartphone linkage has yet another critical function that we have not explored here but demonstrated in prior work [56], which is that of reporting test results, location, and time to the cloud, enabling health officials and policymakers to track the spread of infections, identify vectors, predict spread trajectories, and allocate resources.

To address the needs of resource-limited settings, we also demonstrated low-cost, electricity-free, homemade devices that do not require electronic components and can be produced anywhere. Our homemade devices rely on an exothermic chemical reaction between magnesium alloy and salt water to produce heat, like in Meals Ready to Eat (MRE), and a phase change material to buffer the incubation temperature. Our electricity-free devices are suitable for use in settings with limited infrastructure and during emergencies such as natural disasters, conflicts, or civil unrest, when electricity may be unavailable. For thermal insulation, we used commercially available thermos (~$2) which is considerably lower compared to other rapid diagnostic devices, which are priced between $75 and $5000 [51]. Alternatively, the thermos can be replaced with a Styrofoam cup, lowering costs further and making our entire system disposable and environmentally friendly. Our self-heated device can be linked to a smartphone for data acquisition, processing, and surveillance [53,56]. To the best of our knowledge, this study represents the first application of chemical heating for the detection of poultry viruses. Compared to previously reported chemical heating approaches [48], our method employs EPCM wrapped with Kimwipe paper in a cigarette-style format. This design facilitates water transport via capillary action, effectively mitigating the hydrophobic nature of the PCM and reducing the ramp-up time to less than 5 min.

Our RT-LAMP assays for ILTV, IV (H5N1), and IBV demonstrated analytical sensitivity comparable to previously developed LAMP assays [19]. Chickens infected with ILTV, IV (H5N1), or IBV at early stages of infection typically harbor more than 10^3^ genome copies/μL in respiratory tissues and secretions [23,57,58,59,60]. Thus, our assays are well-suited for early-stage virus detection without the need for target concentration. Specificity was confirmed using a panel of avian viruses, consistently showing high selectivity for target pathogens and accurate detection in clinical samples. Moreover, the assays can be completed in under one hour, offering a rapid alternative to conventional PCR. In terms of cost, each reaction is approximately $2.20 when reagents are purchased in small quantities, and likely much less when purchased in bulk, which is lower than the per-sample cost of other rapid diagnostic tests, ranging from $4 to $75 [51].

To assess the clinical applicability of our LAMP assays and ability to operate with raw samples, we evaluated 33 contrived samples (oropharyngeal swabs spiked with viruses). These included ten ILTV swabs, ten IBV swabs, ten IV swabs, and three negative control swabs. Our results were consistent with the gold standard PCR. If necessary, sample nucleic acid isolation and purification can be carried out at the point of need with portable devices such as slider cassettes [61]. Our methods can be expanded to test other infectious diseases in poultry, other farm animals, and humans at the point of need [49,62]. Further studies using ~100–200 reference and clinical samples covering a wide range of viral loads, geographic locations, and infection stages are essential to validate the performance of our developed assays and devices, along with comparisons to existing commercially available tests like the Alveo Sense ^TM^ Poultry Avian Influenza Test [47]. In our experiments, we used liquid reagents that require refrigeration. These can be readily replaced with lyophilized RT-LAMP and LAMP reaction mixes available from various vendors with 12 months or longer shelf life at ambient temperatures below 32 °C.

Future work may modify the assays to distinguish between vaccine and virulent strains through more specific primers or other means to improve control strategies. Additionally, simple and user-friendly nucleic acid extraction, concentration, and purification methods are sorely needed for on-site diagnostics.

## Data Availability

The data supporting this study’s findings are available from the corresponding author upon reasonable request.

## Supplementary Materials

The following supporting information can be downloaded at https://www.mdpi.com/article/10.3390/bios15100638/s1, Table S1: Synthesized AIV-Matrix Protein Gene cDNA (236 bp) based on a reference H5N1-AIV (Accession number: AM911067.1); Table S2: Sequences and concentrations of ILTV and IBV LAMP primers; Table S3: Data used for Probit analysis to estimate the limit of detection (LOD) of our LAMP assays using both the EzDx device and the electricity-free device; Table S4: qPCR threshold cycle values of clinical samples determined by thermocycler; Figure S1: Step-by-step preparation of the EPCM. (A) Preparing a mixture of Mg/Fe alloy and PCM to form EPCM and distributing the mixture on a Kimwipe tissue. (B) The Kimwipe paper is folded to envelope the EPCM. (C) The Kimwipe paper with EPCM is rolled cigarette style to form the heating canister. (D) The heating canister is placed in the amplification chamber of our electricity-free device; Figure S2: The temperatures of calibration test tubes as functions of time when incubated with fuel separated from PCM and with EPCM. The room temperature is 22 °C.
