# Supplementary material for: Smartphone-Linked and Electricity-Free Platforms for Rapid Colorimetric Molecular Detection of Poultry Respiratory Viruses at the Point of Need
Source: Biosensors (Basel). 2025 Sep 24;15(10):638. doi: 10.3390/bios15100638 (PMC12562839; doi:10.3390/bios15100638)
Supplement: Supplementary file 1 [file biosensors-15-00638-s001.zip › biosensors-3883553-supplementary.pdf]

Supplementary Materials

Smartphone-Linked and Electricity-Free Platforms for Rapid Colorimetric Molecular Detection of Poultry Respiratory Viruses at the Point of Need

Mohamed El-Tholoth [1,2,*], Rabiha Seboussi [3], Mahmoud Hussein [3], Salameh Rahmdel [1], Alanoud Alalawi [1] and Haim H. Bau [4]

1 Health Sciences Division, Al Ain Zakhir Campus, Higher Colleges of Technology, Abu Dhabi 17155, United Arab Emirates

2 Department of Virology, Faculty of Veterinary Medicine, Mansoura University, Mansoura 35516, Egypt

3 College of Veterinary Medicine, University of Al Dhaid, Sharjah, United Arab Emirates

4 Department of Mechanical Engineering and Applied Mechanics, University of Pennsylvania, Philadelphia, PA 19104, USA

* Correspondence: tholothvirol@mans.edu.eg or meltholoth@hct.ac.ae

Table S1: Synthesized AIV-Matrix Protein Gene cDNA (236 bp) based on a reference H5N1-AIV (Accession number: AM911067.1).

**Sequence (5' to 3')**
TCGAAACGTACGTTCTCTCTATCATCCCGTCAGGCCCCCTCAAAGCCGAGATCGCGCAGAAACT<br>TGAGGATGTCTTTGCAGGAAAGAACACCGATCTCGAGGCTCTCATGGAGTGGCTAAAGACAAG<br>ACCAATCCTGTCACCTCTGACTAAAGGGATGTTGGGATTTGTATTCACGCTCACCGTGCCCAGTG<br>AGCGAGGACTGCAGCGTAGACGCTTTGTCCAAAACGCCCTAAAT

Table S2: Sequences and concentrations of ILTV and IBV LAMP primers.

Virus (Target Gene)	Primer	Primer sequences (5' to 3')	Concentration (mM)	Reference
ILTV	F3	GTGGACGACATGATATCGG	0.2	1
	B3	TTCAGGGTCAGCAAGTATTG	0.2	
	FIP (F1c +F2)	CGCCACATTCCCTGTACTCTTTTTCTGGTTCT ACGTGATTAAGG	1.6	
	BIP (B1c +B2)	TGCTATCTGAATGCGCCGTTTACAAGGGTGC TAGGAACA	1.6	
	Loop F	GTACTTGTCCTCGCCGTC	0.8	
	Loop B	AGATGTGGGCAGTGGACTA	0.8	
IBV	F3	CCACCTGGTTATAAGGTTGA**Y**C	0.2	2
	B3	CTACACCATCGAC**R**CACTG	0.2	
	FIP (F1c +F2)	CATGGCTGCTAGGGACCAGATGACAAGATG AATGAGGA**R**GG	1.6	
	BIP (B1c +B2)	TGACGCCCAAACTTCAACCAACGTGACACCA CAGTAGTA	1.6	
	Loop F	GAGCATTGCTGTAACACGC	0.8	
	Loop B	TGGGCTGCACTTGAAATTTG	0.8	
IV	F3	TCGAAACGTACGTTCTCTCT	0.2	This Study
	B3	ATTTAGGGCGTTTTGGACAAAG	0.2	
	FIP (F1c +F2)	CCCGTCAGGCCCCCTCAAAGACCGATCTCGA GGCTCTCATG	1.6	
	BIP (B1c +B2)	TGCAGCGTAGACGCTTTGTCCCAATCCTGTC ACCTCTGACTA	1.6	
	Loop F	GAATGGCTAAAGACAAGACC	0.8	
	Loop B	CACGCTCACCGTGCCCAGTG	0.8	

Y: C/T ; **R:** A/G

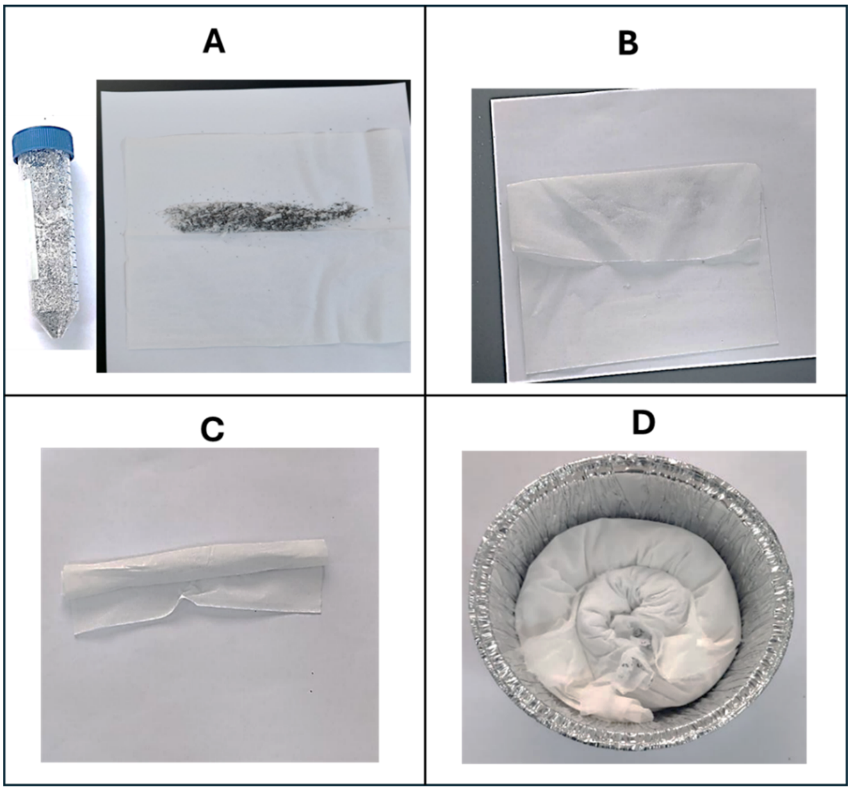

Supplementary Figure S1: Step-by-step preparation of the EPCM. (A) Preparing a mixture of Mg/Fe alloy and PCM to form EPCM and distributing the mixture on a Kimwipe tissue. (B) The Kimwipe paper is folded to envelope the EPCM. C) The Kimwipe paper with EPCM is rolled cigarette style to form the heating canister. D) The heating canister is placed in the amplification chamber of our electricity-free device.

Table S3: Data used for Probit analysis to estimate the limit of detection (LOD) of our LAMP assays using both the EzDx device and the electricity-free device.

Virus	Genome Concentration (genome copies/ reaction)	EzDx Wed Pro-1 (Number positive/tested)	Electricity free device (Number positive/tested)
ILTV	250	8/8	8/8
	200	8/8	7/8
	150	1/8	1/8
	100	0/8	0/8
	50	0/8	0/8
	0	0/8	0/8
IBV	400	8/8	8/8
	350	8/8	8/8
	300	8/8	8/8
	250	2/8	1/8
	200	0/8	0/8
	100	0/8	0/8
	50	0/8	0/8
	0	0/8	0/8
IV	50	8/8	7/8
	25	0/8	0/8

	10	0/8	0/8
	5	0/8	0/8
	0	0/8	0/8

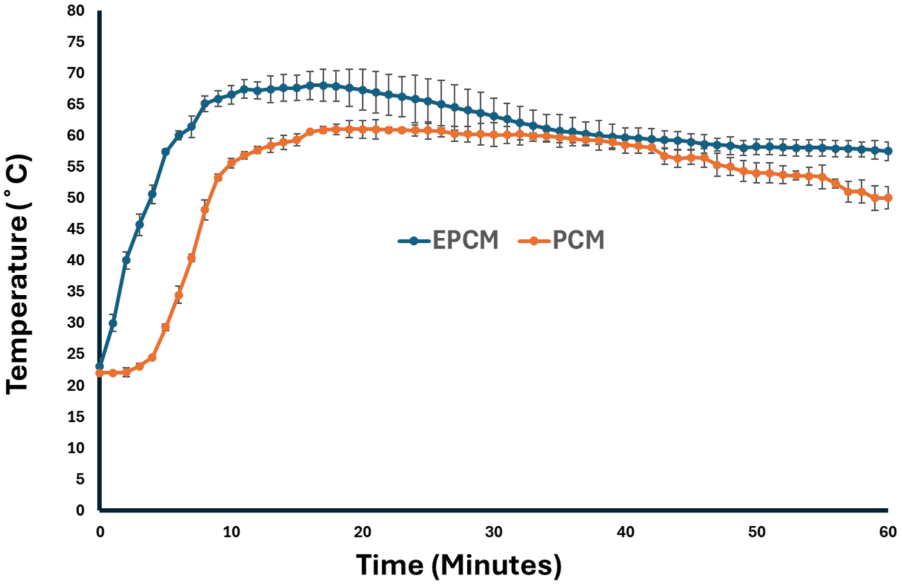

Supplementary Figure S2: The temperatures of calibration test tubes as functions of time when incubated with fuel separated from PCM and with EPCM. The room temperature is 22°C.

Table S4: qPCR threshold cycle values of clinical samples determined by thermocycler.

Sample no	qPCR
ILTV- 1	19.80
ILTV- 2	27.15
ILTV- 3	24.00
ILTV- 4	22.71
ILTV-5	19.00
ILTV- 6	23.51
ILTV- 7	18.04
ILTV- 8	19.31
ILTV- 9	25.78
ILTV- 10	23.62
IBV-1	29.15
IBV-2	34.33
IBV-3	35.00
IBV-4	22.05
IBV-5	23.65
IBV-6	23.46
IBV-7	27.03
IBV-8	29.15
IBV-9	34.33
IBV-10	35.00
IV-1	18.01
IV-2	17.4
IV-3	19.23
IV-4	24.45
IV-5	19
IV-6	20.33
IV-7	25.67
IV-8	22.23
IV-9	21
IV-10	18
-ve Samples	0

References

1. El-Tholoth M, Mauk MG, Anis E, Bau HH. A closed-tube, single-step, real time, reverse transcription-loop-mediated isothermal amplification assay for infectious bronchitis virus detection in chickens. J Virol Methods. 2020;284:113940.

2. El-Tholoth M, Bai H, Mauk MG, Anis E, Bau HH. Molecular Detection of Infectious Laryngotracheitis Virus in Chickens with a Microfluidic Chip. Animals (Basel). 2021 Nov 9;11(11):3203.
